# 865. Social Media Secret Facebook Groups for HIV Pre-Exposure Prophylaxis Awareness among Female Sex Workers in Cameroon

**DOI:** 10.1093/ofid/ofab466.1060

**Published:** 2021-12-04

**Authors:** Laia Jimena Vazquez Guillamet, Mary Mah Babey, Njah Mercy, Hassanatu Blake, Amy Jasani, Rahel Kyeng, Pius Tih, Eveline Mboh Khan, Jodie Dionne

**Affiliations:** 1 ISGlobal, Terrassa, Catalonia, Spain; 2 Cameroon Baptist Convention Health Services, Bamenda, Nord-Ouest, Cameroon; 3 University of Alabama at Birmingham, Birmingham, Alabama

## Abstract

**Background:**

About 25% of Cameroonian female sex workers (FSW) lived with HIV in 2018. PrEP was introduced in Cameroon in 2019, with minimal uptake as of 2021. The goal of this pilot project was to evaluate the potential of a novel social media intervention to raise Pre-Exposure Prophylaxis (PrEP) awareness and complement HIV prevention strategies among FSW, a key risk population.

**Methods:**

From October 2020 to April 2021, sixty adult HIV-negative FSW who owned a phone with internet access joined the study; 40 in the intervention arm and 20 in the control arm. The intervention had a Secret Facebook Group (SFG) platform for confidentiality. It included 12 videos on HIV prevention in the local dialect, released over 8 weeks. In-person surveys were administered before and after the intervention, and three months later. Likert scale was used to evaluate the main outcome: PrEP awareness. Data was analyzed using Stata IC/version 14.2.

**Results:**

Demographic characteristics were similar between intervention and control groups for age (29 years, SD7.3), literacy (45% secondary school), parity (1.9, SD1.5), and years as sex worker (7.8, SD5.1). One FSW had heard about PrEP before the intervention. After a brief introduction, 39% (15/38) of FSW in the intervention group and 50% (10/20) in the control group *strongly agreed* to be interested in taking PrEP (p=0.2). Baseline PrEP knowledge was *poor* in the intervention group (15/40, 38%) and *very poor* in the control group (19/20,95%) (p=0.0001). In the second survey, the intervention and control groups’ PrEP knowledge improved (p=0.0001 and p=0.02, respectively). It was more significant in the intervention group, with all FSW reporting *good* level of knowledge (p=0.0001) (Figure 1). In addition, more FSW in the intervention group (67%,27/40) *strongly agreed* to be interested in taking PrEP (p=0.01), while numbers remained similar in the control group (55%, 11/20, p=0.8). Three months after the intervention, 31.5% (12/38) of participants reported *excellent* PrEP knowledge, a significant improvement since the second survey (p=0.02).

Figure 1. Self-reported Pre-Exposure Prophylaxis knowledge before and after intervention in the intervention and control groups.

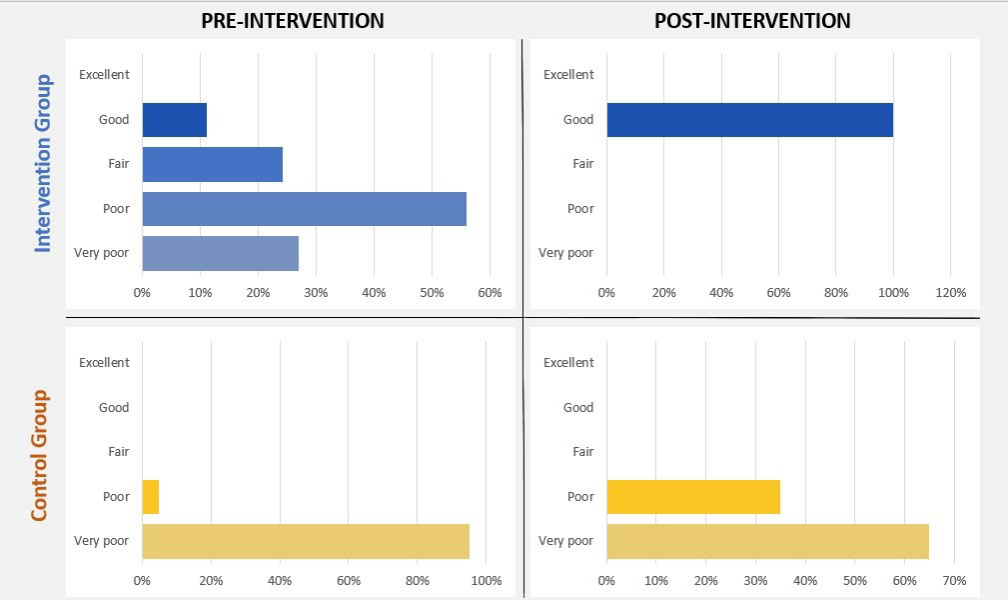

**Conclusion:**

The use of a social media HIV prevention tool tailored to FSW in Cameroon improved PrEP awareness with good retention of knowledge. Cross contamination between groups might have hindered the differential impact of the brief intervention.

**Disclosures:**

**All Authors**: No reported disclosures

